# The CAPRA score versus sub-types of minimal residual disease to predict biochemical failure after external beam radiotherapy

**DOI:** 10.3332/ecancer.2020.1042

**Published:** 2020-05-12

**Authors:** Nigel P Murray, Socrates Aedo, Cynthia Fuentealba, Eduardo Reyes, Anibal Salazar, Eghon Guzman, Shenda Orrego

**Affiliations:** 1 Faculty of Medicine, University Finis Terrae, Providencia, Santiago, 7501015, Chile; 2 Department of Medicine, Hospital de Carabineros de Chile, Ñuñoa, Santiago, 7770199, Chile; 3 Department of Urology, Hospital de Carab Carabineros de Chile, Ñuñoa, Santiago, 7770199, Chile; 4 Faculty of Medicine, University Diego Portales, Santiago, 8370179, Chile; 5 Urology Service, Hospital DIPRECA, Las Condes, Santiago, 7601003, Chile

**Keywords:** prostate cancer, biochemical failure, CAPRA score, minimal residual disease, circulating tumour cells, micro-metastasis

## Abstract

**Introduction:**

External beam radiotherapy is a treatment option for clinically localised prostate cancer; however, some 15% of patients will undergo treatment failure within 5 years. The objective was to compare the Cancer of the Prostate Risk Assessment (CAPRA) score (based on the clinical-pathological findings) and the sub-types of minimal residual disease (MRD) (based on the biological properties of the cancer cells) risk classifications to predict biochemical failure (BF) after external beam radiotherapy.

**Methods and Patients:**

Clinical-pathological findings were obtained from the prostate biopsy to determine the CAPRA score and used to define low-, intermediate- and high-risk patients. Blood and bone marrow were obtained 3 months after radiotherapy; circulating prostate cells (CPCs) and micro-metastasis were detected using immunocytochemistry with anti-prostate specific antigen. CPCs and micro-metastasis were classified as positive if at least one cell was detected in the sample. Three subgroups were formed Group A (MRD negative), Group B (micro-metastasis positive, CPC negative) and Group C (CPC positive)

Patients were followed up for 10 years or until biochemical failure. Biochemical failure free survival (BFFS) curves were constructed using Kaplan–Meier (observed), a flexible parameter model (predicted survival) and the restricted mean survival time (RMST) was calculated for each sub-group.

**Results:**

309 men participated with a median follow-up of 8 years. The risk of biochemical failure increased proportionally with increasing CAPRA score, hazard ratio 1.18 for intermediate and 1.69 for high risk patients. After 10 years, the percentage BFFS and RMST to failure were 74%, 49%, 16% and 9, 7 and 6 years, respectively. The agreement between observed and predicted BFFS was acceptable (Harrell´s C 0.62). The BFFS curves for MRD were different and not proportional, survival curves for men MRD negative and only micro-metastasis were similar up to 5 years, and then there was increasing failure in the micro-metastasis only group. After 10 years, the percentage BFFS and RMST to failure were 95%, 59%, 28% and 10, 9 and 6 years, respectively. The CAPRA score failed to distinguish between Groups A and B, one third of high risk Group C had low risk CAPRA scores. The agreement between observed and predicted BFFS was very good (Harrell´s C 0.92). Minimal residual disease hazard ratios were Group B 1.84 and Group C 4.51.

**Conclusions:**

The MRD prognostic classification is based on the biological characteristics of the tumour cell-microenvironment interaction, to give three groups, MRD negative, only bone marrow micro-metastasis and CPC positive prostate cancer. Differing from the CAPRA score classification the risk of treatment failure changes with time, differentiating between early and late treatment failures and incorporates the concept of dormancy. It proved to be superior to the CAPRA score in predicting biochemical failure and the results need to be confirmed in larger studies.

## Introduction

External beam radiotherapy is one of the treatment options for clinically localised prostate cancer; however, some 10%–15% of patients will undergo treatment failure within 5 years [1]. Pre-treatment prostate specific antigen (PSA) levels, Gleason score, clinical stage and percentage of prostate biopsy cores positive for cancer have all been reported to be independent prognostic factors. Mathematically-based predictive models have been developed using these prognostic factors to improve pre-treatment outcome prediction. The University of California, San Francisco Cancer of the Prostate Risk Assessment (CAPRA) score is one such model [2]. Although initially designed for patients treated by radical prostatectomy, it has been reported to be useful in patients treated with external beam radiotherapy (EBRT) [3–5]. The CAPRA score divides patients into three risk groups, low, intermediate and high risk of biochemical failure. It has been used to define treatments, in that low risk patients can be treated with radiotherapy alone, high risk patients are candidates for androgen deprivation therapy (ADT) after radiotherapy. For intermediate risk patients, the benefit of ADT after radiotherapy has been questioned [6] and as such newer risk classifications to help in clinical decision-making about patient management are important.

Using three-dimensional conformal EBRT with dose of greater than 76 Gy as mono-therapy, biochemical failure free survival rates for low risk patients have been reported to be 94% and 81% at 5 and 10 years, respectively, and 86% and 71% for intermediate risk patients [7]. The Phoenix criteria of ‘American Society for Therapeutic Radiology and Oncology (ASTRA II)’ of a PSA serum level of 2 ng/ml over the PSA nadir obtained after radiotherapy being used to define treatment failure [8].

Treatment failure arises from the proliferation of tumour cells not eradication by curative therapy, these micro-metastasis not detected by conventional studies are termed minimal residual disease. We have recently described two sub-types of minimal residual disease (MRD), those patients with circulating prostate cells detected in blood (independent of whether there are micro-metastasis detected in the bone marrow or not) have a high risk of early treatment failure, while patients only positive for bone marrow micro-metastasis are at risk for late failure and have a similar outcome to MRD negative patients for the first 5 years [9, 10].

The objective of this study was to compare the CAPRA score and MRD prognostic classification to assess the risk and time to biochemical failure in patients treated with EBRT mono-therapy for prostate cancer.

## Patients and methods

We conducted a prospective, observational single centre study of men who underwent EBRT as the sole treatment for prostate cancer between the years 2000 and 2010. The study was approved by the local ethics committee and complied with the Declaration of Helsinki.

For each patient, after giving informed written consent, the following were recorded; date of EBRT, age, serum total PSA (ng/ml) at the time of diagnosis using the Siemens Advia CentaurXR system, clinical status based on digital rectal examination according to the TNM classification system from 1997 [11], and Gleason score and percentage of the prostate biopsy infiltrated by cancer was determined by a single uro-pathologist. The study commenced in 2000 and as such the 2005 Gleason score modifications [12] classifying Gleason 7 as 3 + 4 and 4 + 3 were incorporated. Patient biopsied before 2006 had the biopsies re-evaluated and re-classified according to the 2005 criteria. From these data, the CAPRA score for each patient was calculated as originally described [2].

All of the patients received 3D conformal radiation therapy in daily fractions of 2 Gy, 5 days a week, without boosters, for an average dose of 75 Gy to the prostate (range: 74.1–76 Gy). During the follow-up, the level of total serum PSA was measured every 3 months for the first 2 years and then every 6 months until biochemical failure occurred or until the last control. Biochemical failure was defined as an increase of more than 2 ng/ml above the nadir level of serum PSA obtained after completing EBRT according to the ‘Phoenix’ criteria of ASTRA II [8].

### Detection of minimal residual disease

#### a) Detection of circulating prostate cells

Three months after completing EBRT an 8 ml venous blood sample was taken using ethylendiaminotetraacetic acid as the anticoagulant (BD Vacutainer, USA). Samples were maintained at room temperature and processed within 24 hours. Mononuclear cells were obtained using differential gel centrifugation with Histopaque 1.077 (Sigma-Aldrich, USA) according to the manufacturers’ instructions. The cells were washed using phosphate buffered saline (PBS) pH 7.4 and re-suspended in 100 ml of autologous plasma. 25 ml of cell suspension were used to make four slides (sialinised, DAKO, USA), these were air dried for 24 hours and finally fixed using a solution of 70% ethanol, 5% formaldehyde and 25% PBS pH 7.4 and washed with PBS.

**Figure 1.**
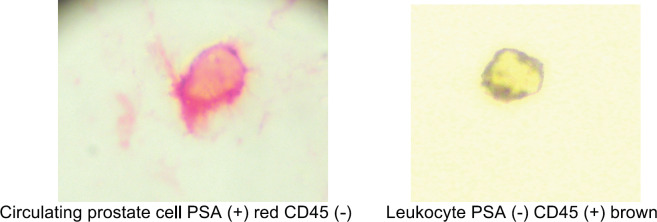
Circulating prostate cell and leukocyte.

Circulating prostate cells (CPCs) were detected using immunocytochemistry with anti-PSA clone 28A4 (DAKO, USA) and identified with an alkaline phosphatase-anti alkaline phosphatase based commercial kit (LSAB2, DAKO, USA) with new fuschin as the chromogen. Samples with cells staining for PSA underwent a second process with anti-CD45 (pan-leukocyte) (DAKO, USA) and identified with a peroxidase-based commercial kit (LSAB2, DAKO USA with DAB (3, 3 diaminobenzidine tetrahydrochloride) as the chromogen. The samples were analysed manually by a single immunocytologist who was blinded to the clinical data. The International Society of Hemotherapy and Genetic Engineering guidelines [13] were used to define a CPC, as a cell expressing PSA but not CD45, whereas a leukocyte expressed CD45 but not PSA (Figure 1). A test was considered to be positive if one CPC was detected and the total number of CPCs/8 ml blood sample was registered.

#### b) Detection of bone marrow micro-metastasis

**Figure 2.**
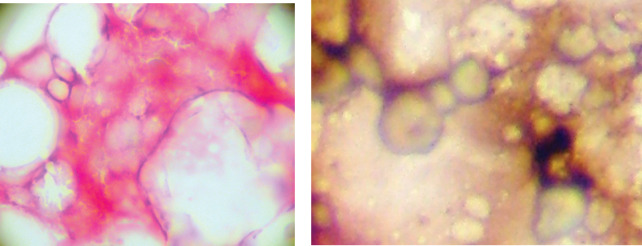
Bone marrow micro-metastasis positive and negative.

At the same time, as the blood sample was taken a bone marrow biopsy was taken from the posterior superior iliac crest using midazolam as sedation and lidocaine as local anaesthetic. Prostate cells detected in bone marrow aspirates are phenotypically different from those detected in bone marrow biopsies, and may represent circulating tumour cells rather than ‘true’ micro-metastasis [14]. Four ‘touch-preps’ using sialinised slides (DAKO, USA) from the biopsy core and were processed as described for CPCs. A micro-metastasis was defined as cells staining positive for PSA and negative for CD45 and classified as positive if one or more cells staining for PSA were detected or negative (Figure 2).

### Classification of patients

The patients were divided into three CAPRA risk groups, low (CAPRA 0-2), intermediate (CAPRA 3-5) and high risk (CAPRA 6-10). Patients were also divided into three MRD prognostic subgroups; Group A: negative for both CPCs and micro-metastasis, Group B: CPCs negative but micro-metastasis positive and Group C: CPCs positive with or without micro-metastasis detected.

### Statistical analysis

The program Stata/SE 16.0 for Windows (Stata Corp LLC) was used to perform the statistical analysis. The quantitative and ordinal variables according to the nature and distribution were described with respective central tendency and dispersion measurements [15]. The nominal variables were described as proportions with their respective confidence intervals [15]. In this description, the subjects were divided into three MRD prognostic groups A, B and C as previously was described.

The prognostic groups were compared for age, total serum PSA, biopsy Gleason score, clinical stage and percentage of biopsy cores infiltrated with cancer. The Marascuillo procedure and Fishers’ Exact tests were used for comparing multiple proportions. The Kruskal–Wallis test was used to test whether samples originate from the same distribution. A *p* value <0.05 was taken to signify statistical significance and all tests were two tailed [15].

In the whole cohort and by MRD prognostic and CAPRA Score groups, a nonparametric biochemical failure free survival analysis was performed at ten years of follow-up, establishing the biochemical failure free survival proportion of Kaplan–Meier and Restricted Mean Survival Time (RMST) [15, 16]. The RMST to 10 years establishes the expected time to the event during 10 years of observation and its value is the area under the Kaplan–Meier nonparametric survival curve [16]. A non-parametric comparison (test Log-Rank) of the biochemical failure free survival by MRD prognostic and by CAPRA score groups was performed. [15, 16]

Multivariable survival analyses are generally carried out using Cox regression. However, several authors have highlighted the limitations of this method for prognostic models, particularly relating to the appropriate modelling of the baseline hazards function [17, 18]. According to the proposed hypothesis with ‘dormant minimal residual disease’, there should be a period of time where the prognosis groups A and B should show a similar biochemical failure free survival curve; at some time later, the biochemical failure free survival curves separate with Group B patients showing a worse survival (CPCs negative micro-metastasis positive). This situation breaches the assumption of proportional risks for use the Cox regression model [15, 16] and as such cannot be used.

An alternative to the Cox model, known as a flexible parametric survival model (FP model), permits the prediction (not descriptive like Kaplan–Meier model) of survival when there is no compliance with the proportional risk’s assumption [17–19].

The FP model should be understood as a regression method in which the dependent variable is the survival for the studied outcome. This method uses the transformation of the independent variable (restricted cubic splines) and its iteration respective with time [17, 19]. Transformations of the independent variables generate different FP models. The degrees of freedom (DF) and the degrees of freedom for each time-dependent effect (DFTVC) indicate the transformations (number of knots) of the independent variables [17, 19].

On assessment of the prediction of biochemical failure, for a follow-up time of 10 years by MRD prognostic groups, a first FP model was built considering the following dummy independent variable: CPCs negative and micro-metastasis positive (prognostic group B) and CPCs positive (prognostic group C).

On assessment of the prediction of biochemical failure, for a follow-up time of 10 years by CAPRA score groups, a second FP model was built considering the following independent variables: CAPRA score between 3 and 5 (CAPRA score Group 2) and CAPRA score between 6 to 10 (CAPRA score Group 3)

The criterion for the selection of predicted model for each of the two FP models built were performed on based the likelihood (less than 0.05) and Bayesian and Akaike criteria, which determine the best adjustment [19].

The calibration aspect of the model refers to agreements between the predicted outcome and observed outcome [20]. We assessment the calibration in the two FP predicted model by graphics comparing predicted FP survival model and observed Kaplan–Meier survival model.

The discrimination of a prognostic model reflects its ability to distinguish between patient outcomes. We assessment the discrimination on the two FP predicted model using the Harrell’s C discrimination index [20],

From the FP predicted biochemical failure, free survival model for up to 10 years, the RMST and survival proportion were determined for each prognostic group of MRD and similarly for the CAPRA subgroups.

The decision curve analysis [21] is a method to evaluate and compare prediction models and to determine the clinical consequences that is treated or not treated. A decision curve analysis was performed for the two predictive models, comparing the clinical utility of survival of the prognostic groups and CAPRA groups.

## Results

A total of 641 subjects were recruited, of these 309 men underwent EBRT as mono-therapy. Thus, the observed cohort included 309 men, whose follow-up time showed a median of 8.03 years with interquartile range (IQR) of 4.61 years. The follow-up time showed a minimum and maximum, respectively, 0.8 and 15.3 years.

The age showed symmetrically distributed with mean ± standard deviation of 68.1 years ± 5.7 years. The serum total PSA showed asymmetrically distributed with median and IQR respective of 5.17 and 2.26 ng/ml.

Table 1 shows the comparison between the MRD prognostic groups. There were significant differences in the serum PSA, Gleason score and CAPRA score between groups A versus C and B versus C. The clinical stage showed significant difference between groups: A versus B, A versus C and B versus C. There was no significant difference in the distribution of CAPRA scores between MRD Group A and B patients, both differing significantly from MRD Group C patients where there was a significantly higher number of high risk CAPRA scores. However, even in CPC positive patients, over one third were classified as low risk using the CAPRA score.

After 10 years of follow up, the observed Kaplan-Meier biochemical failure free survival (BFFS) and RMST (area under the Kaplan–Meier nonparametric survival curve) according to MRD prognostic groups and CAPRA score groups are shown in Table 2. The Log-Rank Test showed a *p*-value less than 0.01 comparing the BFFS between the MRD prognostic groups and the CAPRA score groups. There are significant differences between the two classification systems, in the CAPRA classification with increasing risk score the BFFS and RMST decreases. This differs from the MRD classification in that although with increasing risk group the BFFS decreases, the RMST for Group A and B are similar.

The FP survival model for prediction of biochemical failure at ten years by MRD prognostic groups, showed two degrees of freedom for the restricted cubic spline function used for the baseline hazard rate (DF2). This incorporated the following coefficients: a) CPCs negative and micro-metastasis positive (prognostic group B): Hazard ratio 1.84 (*p*-value < 0.01) and b) CPCs positive (prognostic group C): Hazard ratio 4.51 (*p*-value < 0.01).

The FP survival model for prediction of biochemical failure at ten years by CAPRA score groups, showed one degree of freedom for the restricted cubic spline function used for the baseline hazard rate (DF1). This incorporated the following coefficients: a) CAPRA score between 3 and 5 (CAPRA score group 2): Hazard ratio 1.18 (*p*-value < 0.01) and b) CAPRA score between 6 and 10 (CAPRA score group 3): Hazard ratio 1.69 (*p*-value < 0.01).

**Figure 3.**
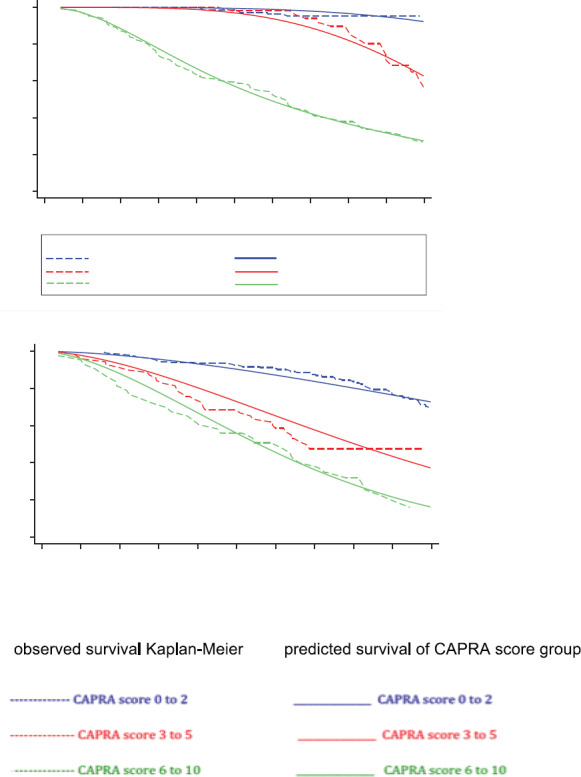
Observed biochemical failure free survival curves (Kaplan–Meier) versus predicted biochemical failure free survival curves for FP model by MRD prognostic groups and for FP model by CAPRA score groups in 311 men with and without biochemical failure treated by EBRT for prostate cancer followed for 10 years. MRD = minimal residual disease; FP = flexible parametric; CPCs = secondary circulating prostate cells ;mM= micro-metastasis; * Predicted FP model that incorporating: mM positive and CPCs negative (prognostic group B), CPCs positive (prognostic group C) with two degrees of freedom for the restricted cubic spline function used for the baseline hazard rate (DF2) and also, consider the CPCs positive (prognostic group C); as time-dependent effect using one degree of freedom for its fit in model (DFTVC1); ** Predicted FP model that incorporating: CAPRA score between 3 and 5 (CAPRA score group 2), CAPRA score between 6 and 10 (CAPRA score group 3) with one degrees of freedom for the restricted cubic spline function used for the baseline hazard rate (DF2)

There was agreement comparing the FP predictive model with the observed survival (Kaplan–Meier) for MRD prognostic groups with a Harrell´s C index of 0.91 (considered very good). There was agreement comparing the predictive and observed survival for the CAPRA groups with a Harrell´s C index of 0.62 (considered acceptable). (Figure 3, Table 2). Table 2 shows the BFFS and the RMST for the differing MRD prognostic groups and CAPRA scores. Figure 3 highlights the difference between the two classifications; with the CAPRA score the three curves are proportional with decreasing BFFS and decreasing RMST with increasing CAPRA score. The curves for the MRD classification are significantly different, in that patients in Group B (CPC negative micro-metastasis positive) have a similar BFFS curve to those patients MRD negative (Group A) for the first five years, thereafter there is a divergent pattern with increasing biochemical failure in Group B patients.

**Figure 4.**
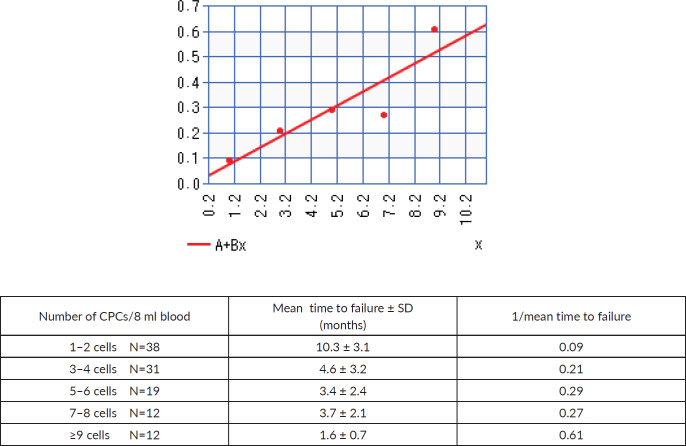
Linear regression comparing the number of CPCs detected versus 1/time to biochemical failure.

Figure 4 shows the linear regression curve comparing the number of CPCs detected and the mean time to failure, represented as 1/mean time to failure, with increasing numbers of CPCs detected the time to biochemical failure shortened, with an *r* = 0.9 (strong correlation).

**Figure 5.**
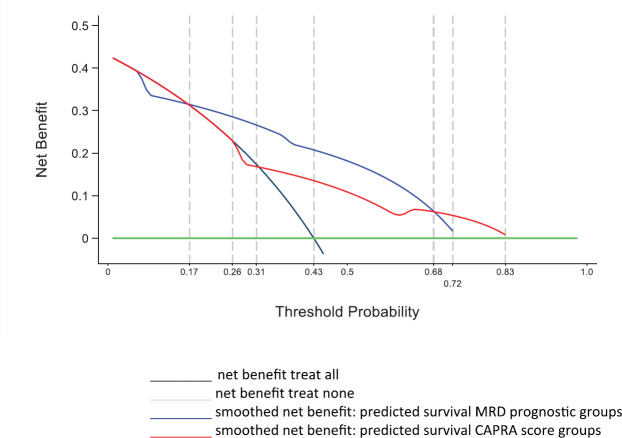
Decision curve analysis for FP model of MRD prognostic groups and FP model of CAPRA score groups in 311 men with and without biochemical failure treated by EBRT for prostate cancer followed for 10 years.

Figure 5 shows the results of the decision curve analysis for the FP model of MRD prognostic groups and FP model of CAPRA score groups for the range of probability threshold values observed between 0 and 1. In men treated by radical prostatectomy and followed for 10 years for a probability threshold of 0.15 to 0.71, the model based on the MRD prognostic groups was superior to the model based on CAPRA score groups. For a threshold probability smaller than 0.15, the CAPRA score model was similar to the strategy treat all. Likewise, for a threshold probability higher than 0.71, the CAPRA score model was similar to the MRD prognostic groups for predicting biochemical failure.

## Discussion

The CAPRA score for predicting future biochemical failure after EBRT has been externally validated and divides patients into risk groups, defined as low, intermediate and high risk. The concordance index (C-index) between predicted BFFS (CAPRA) and observed BFFS (Kaplan-Meier) has been reported to be between 0.62 and 0.66 at 5 years and 0.62 at 8 years [3–5]. The results of our study group showed a C-index of 0.62 at 10 years was similar to that reported in the literature. The observed BFFS rates at 5 years for the three groups are also similar to the published data [3–5]. The CAPRA score is based on the pathological findings in the prostate biopsy combined with the pre-surgical serum PSA and age as such the score is a combination of known risk factors associated with future failure. However, not all cancer cells are equal; there is heterogeneity in the phenotypic expression of tumour cells in the same patient. Subpopulations of tumour cells are capable of disseminating early in prostate cancer [23]. Not all cancer cells are capable of active dissemination, survival in the circulation, extravasation and survival in distant tissues. The morphological characteristics used to define Gleason score do not identify these characteristics. Especially, in Gleason 7 patients (3 + 4 and 4 +3), there is heterogeneity in clinical outcomes, more recently genomic testing have revealed differences that predict indolent Gleason 7 cancers from aggressive lethal ones [24, 25]. Within the same patient, there is considerable variability in genomic alterations found in biopsy cores [26] and that most primary prostate cancers consist of multiple tumours within the same organ and these different tumours rarely share somatic gene mutations or cancer driver genes [27]. The parameters used to determine the CAPRA score are fixed, thus changes with time in the biological characteristics of disseminated tumour cells in blood and/or bone marrow will not be reflected in the risk score. In this study the timing of sampling for MRD detection was fixed, but the method permits repeat sampling during follow-up. This could be important in that the appearance of CPCs in patients previously only positive for micro-metastasis could signal a change in disease progression and predict impending biochemical failure.

Tumour cells that have disseminated will be outside of the radiation field and as such not treated, these micro-foci of tumour cells are called MRD. We have previously reported that there are at least two subtypes of MRD; firstly, those patients with CPCs detected in the circulation and secondly those patients with only bone marrow micro-metastasis. The presence of CPCs independent of whether bone marrow micro-metastasis are present or not is associated with an increased risk of early failure, while those with only bone marrow micro-metastasis are at risk of late failure [9]. This is independent of whether the patient is treated with EBRT or prostatectomy [9]. Here, we report that the mean time to biochemical failure significantly decreases with increasing number of CPCs detected. The limited number of patients based on CPC number subgrouping does not permit a more detailed analysis.

What is important is that in the micro-metastasis group of patients (Group B in this study) for the first 5 years the BFFS is similar to those patients MRD negative. This is explained by the concept of dormancy. The interactions between the tumour cell and microenvironment, including the immune system determines whether tumour cells proliferate or remain in a quiescent state. This quiescent state may last for years and is seen in the clinical situation as the time between primary treatment and failure in patients without evidence of metastatic disease. Changes in the tumour cells, such as clonal progression [28] or changes in immune surveillance, may lead to tumour activation. The CAPRA score was not significantly different between men MRD negative and those micro-metastasis positive (Group B), and thus unlike the MRD prognostic classification was unable to identify those men at risk of late failure. Similarly, the CAPRA score classified patients as having a low risk of treatment failure in the CPC positive high-risk group and thus adjuvant therapy may not be considered.

The risk of biochemical failure in Group B patients changes with time, and thus analysis using Cox proportional hazards model is not applicable. This new prognostic model improves further the predictive value, identifying men with the risk of late failure and who for the first five years appear to be in remission. The Harrell´s C index of the MRD prognostic test was superior to the CAPRA score in predicting biochemical failure free survival.

The clinical usefulness of detecting the sub-types of MRD depends on its ability to bring possible benefits for patients by differentiating the following alternate therapies: early adjuvant or salvage therapy if CPCs are detected, if micro-metastasis are also present the implication is that local radiotherapy will not be sufficient and hormonal therapy may be more beneficial. In patients with only micro-metastasis detected long-term follow up and hormonal therapy as a treatment option at biochemical failure and finally those patients negative for MRD who may require less frequent follow-up. The decision curve analysis used determines the net benefit of a medical decision, which is the difference between the benefit and harms of treatment [29]. In this study, the MRD prognostic evaluation was superior to the CAPRA score. Thus, we consider that the MRD prognostic model gives clinically significant information to aid the decision on who may be eligible for adjuvant therapy, the type of therapy systemic or local, the timing for early or late failure and conversely those patients who may not need adjuvant therapy. The use of MRD detection permits the sequential follow up of patients, detecting changes in the biological characteristics of tumour cells detected in blood and bone marrow, and thus permitting changes in risk classification and/or treatment decisions.

The study has several limitations; the detection of micro-metastasis using bone marrow aspirations or biopsy has been documented although differing antibodies have been used to identify tumour cells, anti-cytokeratin, anti-PSA and anti-prostate specific membrane antigen (PSMA) for prostate cells. The use of reverse transcriptase polymerase chain reaction for PSA and PSMA is reported to have ten times the sensitivity to detect tumour cells. However, detecting every cancer may not be important, patients post allogeneic bone marrow transplantation for leukaemia may have very small numbers of leukaemic cells detected by RT-PCR in bone marrow samples but remain in remission for many years. Furthermore these leukaemia cells may survive for prolonged periods before being eradicated by host defenses [30]. As such ultra-sensitive methods to detect tumour cells may over-estimate clinically important minimal residual disease in patients with solid tumours. The time at which sampling is indicated has not been established; we used three months in this study. This time period was selected as it corresponded to the first traditional control post EBRT, not for any other reason and may not be the most indicated.

We used anti-PSA which is specific for prostate and bone marrow biopsy touch-preps for three main reasons; firstly, the samples do not need to be decalcified or an antigen recuperation process and as such epitopes are not destroyed; secondly, the diagnostic accuracy between touch-preps and biopsy samples is reported to be 84% and a positive correlation of 85% with the biopsy specimen [31]. Finally, the cells detected in bone marrow aspirates may be cells circulating in the bone marrow compartment (equivalent to CPCs) and not true micro-metastasis and are phenotypically different [14]. Although thought to be an invasion procedure, performed under sedation and local anaesthesia, the risk of adverse effects is minimum, less than 0.08% in the British Society of Haematology review of 20,000 procedures [32].

We used differential gel centrifugation and immunocytochemistry for the detection of CPCs, acknowledging that the detection of CPCs or CTCs is method dependent.

In this study, we used the combination of anti-PSA and anti-CD45, the definition of a CPC was that used to define micro-metastasis in previously reported studies. This has a disadvantage that it identifies prostate cells and not if they are benign or malignant. It is has been reported that prostate cells can be detected in patients with benign disease, hyperplasia and prostatitis [33] and as a result of the prostate biopsy [34]. In this context, inflammation post radiotherapy could result in the release of benign prostate cells into the circulation and cause miss-classification as CPC positive. In studies posterior to 2008, we used a combination of anti-PSA and anti-P504S, cells that co-express P504S are thought to be malignant, while those P504S negative are thought to be benign [33–35]. This combination permits the differentiation between benign and malignant cells released into the circulation and such decreases the possibility of false positive results.

However, although the study had the disadvantage of being a single centre, it has the advantage of an immunocytologist who has the experience and training to perform the tests which have been internally validated as to pre-analytical, analytical and post analytical variables as described in the methods section. However, the use of standard immunocytochemistry has the advantage that it could be carried out in the routine laboratory of a general hospital without the need for high cost technology. Immunocytochemical analysis of tissue samples is a part of the routine pathological analysis, such as bone marrow samples and cytology samples, do not represent a novel technique. The only novel procedure is the obtaining CPCs using differential gel centrifugation, in an internal validation of the inter and intra-observer reliability of the CPC determination in 30 subjects analysed in duplicate by three different pathologists, the observed inter-operator agreement was 89% and inter-observer agreement was 90%, with a kappa statistic of 0.77 and 0.79 classified as good agreement [36].

Using the Epithelial Cell Adhesion Molecule-based CellSearch® system, the frequency of patients positive for CPCs has been reported to be between 5% and 42% in patients with localised cancer [37, 38], Comparing three different methods of CPC detection, the CellSearchÒ system detect CPCs in 14% of high risk patients, the EPISPOT assay in 42%Ò of patients and in 48% of patients using the CellCollectorÒ [39]. The differences and pitfalls of the different methods to detect CPCs have been reviewed [40]. The method we used to detect CPCs is based on cell size and density however will not detect CPCs and micro-metastasis that do not express PSA. However, the use of standard immunocytochemistry has the advantage that it could be carried out in the routine laboratory of a general hospital without the need for high cost technology or highly specialised personnel.

The results of the study need to be confirmed with a larger number of patients. However, the presented results show that risk classification based on morphological characteristics may not represent the biological characteristics of a cancer in individual patients, and thus not accurately predict outcome.

## Conclusions

The CAPRA is an externally validated risk classification based on the pre-treatment PSA level, age of the patient and pathological findings in the biopsy specimen. Three risk groups, low, intermediate and high have been identified on which to base treatment decisions and have an acceptable predictive value. The MRD prognostic classification is based on the biological characteristics of the tumour cell-microenvironment interaction, to give three groups, MRD negative, only bone marrow micro-metastasis and CPC positive prostate cancer. Differing from the CAPRA score classification the risk of treatment failure changes with time, differentiating between early and late treatment failures, incorporates the concept of dormancy. The CAPRA does not differentiate between MRD negative and micro-metastasis only prostate cancer patients, and high risk CPC positive patients may be classified as low risk using the CAPRA score. The study results warrant further larger scale confirmation.

## Conflicts of interest

Dr Murray has received consultancy fees from Viatar CTC solutions, Boston, USA.

## Funding

The study was funded by a Hospital de Carabineros de Chile research grant.

## Figures and Tables

**Table 1.** Clinical-pathological features of the prognostic groups on 309 men with and without biochemical failure treated with external beam radiotherapy for prostate cancer followed for ten years.

Characteristic	Group A Absence CPCs Absence mM *n* =139	Group B Absence CPCs Presence mM *n* = 58	Group C Presence CPCs Presence/Absence mM *N* = 112	*p*-value two tail
Age at diagnosis mean± DS	67.1 ; 7.9	69.0 ; 10.2	69.3 ; 8.5	0.232^a^
PSA at diagnosis median; IQR	6.21; 1.52	5.94; 2.12	6.61; 1.62	< 0.01^b^
Gleason score median; IQR:1 ≤6 3 + 4 4 + 3 ≥8	6 122 9 3 5	6; 1 46 6 3 2	7; 2 51 18 16 27	< 0.001^b^ A versus B 0.54^b^
Clinical stage T1 T2 T3	83 48 8	8 43 7	16 58 38	< 0.001^c^ A versus B <0.001^c^
Age < 50 > 50	1 138	0 58	2 110	0.49^d^
% biopsy infiltrated < 34% >34%	64 75	16 42	34 68	0.025^d^ A versus B 0.025^d^ B versus C 0.57^d^ A versus C 0.064^d^
CAPRA 0–2 3–5 ≥6	112 22 12	39 13 9	35 17 50	<0.001^e^ A versus B 0.127^c^ A versus C <0.001^c^ B versus C <0.001^c^

DS = standard deviation; IQR = interquartile range; mM = micro-metastasis; CPC = circulating prostate cell.

a Kruskal–Wallis test;

b Kruskal–Wallis test (significant difference between groups: A versus C and B versus C);

c Kruskal–Wallis test (significant difference between groups: A versus B, A versus C and B versus C);

d Marascuillo procedure (significant difference between groups: A versus C and B versus C);

e Fishers’ Exact tests.

**Table 2.** Observed biochemical failure free survival (Kaplan–Meier) versus predicted biochemical failure free survival for FP model by prognostic groups and for FP model by CAPRA score groups in 309 men with and without biochemical failure treated by EBRT for prostate cancer followed for 10 years.

Type Survival % (95% CI)		Observed^a^		Predicted	
		Survival % (95% CI)	RMST years (95%CI)	Survival % (95% CI)	RMST years (95%CI)
Prognostic group	Group A CPCs and mM negative *n* = 139	94.7 (91.2 to 96.7)	9.7 (9.6 to 9.9)	93.4 ^b^ (82.9 to 96.1)	9.9 ^b^ (9.7 to 9.9)
	Group B CPCs negative and mM positive *n* = 58	58.5 (39.2 to 72.1)	9.4 (9.1 to 9.7)	60.7 ^b^ (44.3 to 76.4)	9.2^b^ (8.6 to 9.6)
	Group C CPCs positive *n* = 112	28.4 (20.1 to 35.4)	5.9 (5.1 to 6.7)	26.2 ^b^ (19.7 to 36.7)	5.9 ^b^ (5.2 to 6.3)
	All subjects *N* = 309	59.2 (52.3 to 64.8)	8.2 (7.7 to 8.5)	63.1 ^b^ (58.85 to 65.12)	8.3 ^b^ (8.0 to 8.6)
CAPRA score groups	Group 1 CAPRA score between 0 and 2 *n* = 186	74.2 (63.6 to 78.4)	9.4 (8.6 to 9.4)	70.3 ^c^ (65.6 to 80.2)	8.6 ^c^ (8.2 to 9.0)
	Group 2 CAPRA Score between 3 and 5 *n* = 52	48.6 (35.2 to 60.4)	6.8 (6.1 to 7.8)	38.4 ^c^ (26.2 to 48.2)	7.1 ^c^ (6.3 to 7.6)
	Group 3 CAPRA score between 6 to 10 *n* = 71	15.7 (6.0 to 28.1)	5.7 (4.7 to 6.6)	15.8^c^ (7.2 to 25.9)	5.7 ^c^ (4.9 to 6.7)
	All subjects *N* = 309	59.03 (50.1 to 63.2)	8.3 (7.6 to 8.6)	59.9 ^c^ (57.1 to 60.3)	8.2 ^c^ (8.0 to 8.4)

FP = flexible parametric; CPCs = secondary circulating prostate cells; mM = micro-metastasis; %: percentage;

a Observed used the Kaplan–Meier survival model;

b Predicted FP model that incorporating: Mm positive and CPCs negative (prognostic group B), CPCs positive (prognostic group C) with two degrees of freedom for the restricted cubic spline function used for the baseline hazard rate (DF2) and also, consider the CPCs positive (prognostic group C); as time-dependent effect using one degree of freedom for its fit in model (DFTVC1);

c Predicted FP model that incorporating: CAPRA score between 3 and 5 (CAPRA score group 2), CAPRA score between 6 and 10 (CAPRA score group 3) with one degrees of freedom for the restricted cubic spline function used for the baseline hazard rate (DF2).

## References

[1] Vassil AD, Murphy ES, Reddy CA (2010). Five-year biochemical recurrence free survival for intermediate risk prostate cancer after radical prostatectomy, external beam radiation therapy or permanent seed implantation. Urol.

[2] Cooperberg MR, Freedland SJ, Pasta DJ (2006). Multi-institutional validation of the UCSF cancer of the prostate risk assessment for prediction of recurrence after radical prostatectomy. Cancer.

[3] Krishnan V, Delouya G, Bahary JP (2014). The Cancer of the Prostate Risk Assessment (CAPRA) score predicts biochemical recurrence in intermediate risk prostate cancer treated with external beam radiotherapy (EBRT) dose escalation or low dose rate (LDR) brachytherapy. BJU Int.

[4] Halverson S, Schipper M, Blas K (2011). The Cancer of the Prostate Risk Assessment (CAPRA) in patients treated with external beam radiation therapy: evaluation and optimization in patients at higher risk of relapse. Radiother Oncol.

[5] Delouya G, Krishnan V, Bahary JP (2014). Analysis of the Cancer of the Prostate Risk Assessment to predict for biochemical failure after external beam radiotherapy or prostate seed brachytherapy. Urol.

[6] Amini A, Rusthoven CG, Jones BL (2016). Survival outcomes of radiotherapy with or without androgen deprivation therapy for patients with intermediate-risk prostate cancer using the National Cancer Data Base. Urol Oncol.

[7] Boladeras A, Martinez E, Ferrer F (2016). Localized prostate cancer treated with external beam radiation therapy: long term outcomes at a European comprehensive cancer center. Rep Pract Oncol Radiother.

[8] Roach M, Hanks G, Thames H (2006). Defining biochemical failure following radiotherapy with or without hormonal therapy in men with clinically localized prostate cancer: recommendations of the RTOG-ASTRO Phoenix Consensus Conference. Int J Radiat Oncol Biol Phys.

[9] Murray NP, Aedo S, Fuentealba C (2019). Circulating prostate cells and bone marrow micro-metastasis and not treatment modality determine the risk and time to biochemical failure in low risk prostate cancer. Arch Esp Urol.

[10] Murray NP, Aedo S, Fuentealba C (2019). Subtypes of minimal residual disease, association with Gleason score, risk and time to biochemical failure in pT2 prostate cancer treated with radical prostatectomy. Ecancermedicine.

[11] Sobin LH, Wittekind CH, International Union against Cancer (UICC) (1997). TNM Classification of Malignant Tumours.

[12] Epstein JL, Allsbrook WC, Amin MB (2005). The 2005 International Society of Urological Pathology (ISUP) Consensus Conference on Gleason Grading of Prostatic Carcinoma. Am J Surg Pathol.

[13] Borgen E, Naume B, Nesland JM (1999). Standardization of the immunocytochemical detection of cancer cells in BM and blood. I. Establishment of objective criteria for the evaluation of immunostained cells. Cytotherapy.

[14] Murray NP, Reyes E, Tapia P (2012). Redefining micro-metastasis in prostate cancer- a comparison of circulating prostate cells, bone marrow disseminated tumor cells and micro-metastasis: Implications in determining local or systemic treatment for biochemical failure after radical prostatectomy. Int J Mol Med.

[15] Rosner B (2015). Fundamentals of biostatistics.

[16] Cleves M, Gutierrez R, Gould W (2010). An Introduction to Survival Analysis using Stata.

[17] Royston P, Parmar MK (2013). Restricted mean survival time: an alternative to the hazard ratio for the design and analysis of randomized trials with a time-to-event outcome. BMC Med Res Methodol.

[18] A´Hern RP (2016). Restricted mean survival time: An obligatory end point for time to event analysis in cancer trials?. J Clin Oncol.

[19] Royston P, Lambert PC (2011). Flexible Parametric Survival Analysis using Stata: Beyond the Cox Model.

[20] Royston P (2015). Tools for checking calibration of a Cox model in external validation: prediction of population-averaged survival curves based on risk group. Stata J.

[21] Choodari-Oskooei B, Royston P, Parmar MK (2012). A simulation study of predictive ability measures in a survival model I: explained variation measures. Stat Med.

[22] Vickers AJ, Cronin AM, Elkin EB (2008). Extensions to decision curve analysis, a novel method for evaluating diagnostic tests, prediction models and molecular markers. BMC Med Inform Decis Mak.

[23] Moreno JG, Croce CM, Fischer R (1992). Detection of hematogenous micrometastasis in patients with prostate cancer. Cancer.

[24] Johnston WL, Catton CN, Swallow CJ (2019). Unbiased data mining identifies cell cycle transcripts that predict non-indolent Gleason score 7 prostate cancer. BMC Urol.

[25] Sinnott JA, Peisch SF, Tyekucheva S (2017). Prognostic utility of a new mRNA expression signature of Gleason score. Clin Cancer Res.

[26] Wei L, Wang J, Lampert E (2017). Intratumoral and intertumoral genomic heterogeneity of multifocal localized prostate cancer impacts molecular classifications and genomic prognosticators. Eur Urol.

[27] Lovf M, Zhao S, Axcrona U (2019). Multifocal primary prostate cancer exhibits high degree of genomic heterogeneity. Eur Urol.

[28] Schutz E, Akbari MR, Beck J (2015). Chromosomal instability in cell-free DNA is a serum biomarker for prostate cancer. Clin Chem.

[29] Vickers AJ, Elkin EB (2006). Decision curve analysis: a novel method for evaluating prediction models. Med Decis Mak Int J Soc Med Decis Mak.

[30] Cross NC (1998). Minimal residual disease in chronic myeloid leukaemia. Hematol Cell Ther.

[31] Chandra S, Chandra H (2011). Comparison of bone marrow aspirate cytology, touch imprint cytology and trephine biopsy for bone marrow evaluation. Hematol Rep.

[32] Bain B (2005). Bone marrow biopsy morbidity: review of 2003. J Clin Pathol.

[33] Murray NP, Reyes E, Badínez L (2013). Circulating prostate cells found in men with benign prostate disease are P504S negative: clinical implications. J Oncol.

[34] Murray NP, Reyes E, Orellana N (2015). Expression of P504S and matrix metalloproteinase-2 in circulating prostate cells disseminated as a result of trans-rectal ultrasound guided biopsy as determined by immunocytochemistry: clinical implications. Arch Esp Urol.

[35] Rubin MA, Zhou M, Dhanasekaran SM (2002). α-methylacyl coenzyme A racemase as a tissue biomarker for prostate cancer. JAMA.

[36] Murray NP, Aedo S, Fuentealba C (2018). Combining the prostate cancer risk index (PRIX) with the presence of secondary circulating prostate cells to predict the risk of biochemical failure after radical prostatectomy for prostate cancer. Asian Pac J Cancer Prev.

[37] Davis JW, Nakanishi H, Kumar VS (2008). Circulating tumor cells in peripheral blood samples from patients with increased serum prostate specific antigen: initial results in early prostate cancer. J Urol.

[38] Eschwège P, Moutereau S, Droupy S (2009). Prognostic value of prostate circulating cells detection in prostate cancer patients: a prospective study. Br J Cancer.

[39] Budna-Tukan J, Swierczewska M, Mazel M (2019). Analysis of circulating tumor cells in patients with non-metastatic high risk prostate cancer before and after radiotherapy using three different enumeration assays. Cancers (Basel).

[40] Panteleakou Z, Lembessis P, Sourla A (2009). Detection of circulating tumor cells in prostate cancer patients: methodological pitfalls and clinical relevance. Mol Med.

